# The Georgia Medical Association

**Published:** 1898-05

**Authors:** 


### THE GEORGIA MEDICAL ASSOCIATION.

The forty-ninth annual meeting of the Georgia State Medical Association was held on Cumberland Island, April 20, 21, 22. The meeting, we are sorry to say, was not up to the usual standard of this Association, especially in the attendance. Cumberland Island is a delightful pleasure resort during the latter part of May or first of June, but as a meeting-place for the State Association in April, we must say that it proved a failure. In the first place, it was too far away from the center of the State, and therefore inaccessible to a good proportion of the members of the Association. It took too long to reach the place, and its distance necessarily made it expensive. The trip to Brunswick might have answered, had the meeting been held in that city, but the reaching of Cumberland Island by means of the water schedule, necessitated the loss of nearly a whole day on the water. Besides, we do not think that a meeting of this kind should be held purely for the sake of pleasure, but accurate scientific and strictly professional labor should be the aim of the State Medical Association. A great many of the members enjoyed the sailing and fishing, which amusement unfortunately proved too much of a drawing card to the detriment of the real work of the meeting. However, we think that the lesson has been taught to consider more seriously in the future the place of the annual meeting.

The final program as printed showed many excellent features, but unfortunately the majority of the papers had to be read by title. The first vice-president, Dr. Hardeman, presided over the meeting with grace and ease. There was a notable absence of the Committee on Arrangements, so that each individual had to seek out his own information. Yet we cannot attribute the seemingly non-success of the meeting entirely to local causes, for our country being deeply stirred by the war measures which have been inaugurated, these have affected the professional man as well as the merchant, and there is no doubt but that this one factor kept many of the members away. However, those papers which were read were good and provoked interesting discussion. The weather was perfect, and the city physicians, especially, seemed to enjoy the outing on the island. The next meeting will be held in Macon, mainly for the reason that the Association will celebrate its semi-centennial anniversary and in that Macon was the home of its inauguration. Some new resolutions were introduced and acted upon which will be seen in the Transactions.

---

DR. WILLIAM H. JOHNSTON, one of the founders and the Dean of the Birmingham Medical College at Birmingham, Ala., died of apoplexy on April 3, in his eightieth year. He was born in Lincoln county, N. C., educated in the State University, and served gallantly in the Twenty-third North Carolina Regiment, Confederate Army. After the war he was graduated in medicine at the University of the City of New York, and served eighteen months at Bellevue Hospital, New York. For a short time he practiced medicine in the latter city, moved to Selma, Ala., in 1872, and to Birmingham in 1886.

